# Supplementary material for: Nature-Based Restoration Simulation for Disaster-Prone Coastal Area Using Green Infrastructure Effect
Source: Int J Environ Res Public Health. 2023 Feb 10;20(4):3096. doi: 10.3390/ijerph20043096 (PMC9960258; doi:10.3390/ijerph20043096)
Supplement: Supplementary file 1 [file ijerph-20-03096-s001.zip › ijerph-2134743-supplementary.pdf]

Supplementary Material

Table S1. Runoff amount via the application of green infrastructure.

Time (hour)	Basic scenario (Green infrastructure 0%)	Scenario 1 (Green infrastructure 10%)	Scenario 2 (Green infrastructure 20%)	Scenario 3 (Green infrastructure 30%)
1	318.5	293.9	269.2	244.6
2	106.2	98.0	89.8	81.6
3	212.4	195.9	179.5	163.1
4	1486.5	1371.7	1256.9	1142.0
5	2336.0	2156.2	1976.5	1796.7
6	4990.5	4608.8	4227.2	3845.5
7	4141.0	3849.1	3557.1	3265.2
8	1380.3	1284.2	1188.0	1091.9
9	6795.5	6324.0	5852.5	5381.0
10	5096.6	4750.0	4403.4	4056.8
11	106.2	99.1	92.0	84.8
12	318.5	297.2	275.9	254.6

Unit of data: m^3

Table S2. Runoff amount via the green infrastructure type in the public area.

Time (hour)	Basic scenario (No green infrastructure)	Scenario 1 (Green roof: GR)	Scenario 2 (Infiltration storage facility: ISF)	Scenario 3 (Porous pavement: PP)
1	37.7	23.1	24.2	32.0
2	12.6	7.7	8.1	10.7
3	25.1	15.4	16.2	21.3
4	176.0	107.7	113.1	149.1
5	276.6	169.3	178.5	234.4
6	591.0	361.7	383.7	500.7
7	490.4	353.1	322.7	415.5
8	163.5	117.7	108.7	138.5
9	804.7	579.4	537.2	681.8
10	603.6	434.6	410.0	511.3
11	12.6	9.1	8.7	10.7
12	37.7	27.2	26.0	32.0

Unit of data: m^3

Table S3. Runoff amount via the green infrastructure type in the private area.

Time (hour)	Basic scenario (No green infrastructure)	Scenario 1 (Green roof: GR)	Scenario 2 (Infiltration storage facility: ISF)	Scenario 3 (Porous pavement: PP)
1	119.2	82.7	85.5	104.9
2	39.7	27.6	28.5	35.0
3	79.5	55.1	57.1	69.9
4	556.5	386.0	399.5	489.4
5	874.5	606.6	629.6	769.0
6	1868.2	1296.0	1351.0	1642.9
7	1550.2	1207.6	1131.6	1363.2
8	516.7	402.5	380.1	454.4
9	2544.0	1981.6	1876.3	2237.1
10	1908.0	1486.2	1425.0	1677.8
11	39.7	31.0	30.0	35.0
12	119.2	92.9	89.9	104.9

Unit of data: m^3

Table S4. Runoff amount via the green infrastructure type in the industrial area.

Time (hour)	Basic scenario (No green infrastructure)	Scenario 1 (Infiltration storage facility: ISF)	Scenario 2 (Porous pavement : PP)
1	33.7	23.6	29.4
2	11.2	7.9	9.8
3	22.5	15.8	19.6
4	157.2	110.4	137.1
5	247.0	174.0	215.5
6	527.6	373.4	460.4
7	437.8	313.0	382.0
8	145.9	105.2	127.3
9	718.4	519.4	626.9
10	538.8	394.8	470.2
11	11.2	8.3	9.8
12	33.7	24.9	29.4

Unit of data: m^3

Table S5. Runoff amount via the green infrastructure type in the Transportation area.

Time (hour)	Basic scenario (No green infrastructure)	Scenario 1 (Infiltration storage facility: ISF)	Scenario 2 (Porous pavement : PP)
1	127.9	89.7	111.6
2	42.6	29.9	37.2
3	85.3	59.8	74.4
4	596.8	419.1	520.8
5	937.9	660.6	818.5
6	2003.6	1418.1	1748.5
7	1662.6	1188.7	1450.9
8	554.2	399.6	483.6
9	2728.4	1972.5	2381.0
10	2046.3	1499.5	1785.7
11	42.6	31.6	37.2
12	127.9	94.7	111.6

Unit of data: m^3
